# Establishing failure predictors for the planned extubation of overweight and obese patients

**DOI:** 10.1371/journal.pone.0183360

**Published:** 2017-08-16

**Authors:** Chien-Ming Chao, Chih-Cheng Lai, Ai-Chin Cheng, Shyh-Ren Chiang, Wei-Lun Liu, Chung-Han Ho, Shu-Chen Hsing, Chin-Ming Chen, Kuo-Chen Cheng

**Affiliations:** 1 Department of Intensive Care Medicine, Chi Mei Medical Center, Liouying, Taiwan; 2 Departments of Internal Medicine, Chi Mei Medical Center, Tainan, Taiwan; 3 Chia Nan University of Pharmacy & Science, Tainan, Taiwan; 4 Departments of Medical Research, Chi Mei Medical Center, Tainan, Taiwan; 5 Departments of Intensive Care Medicine, Chi Mei Medical Center, Tainan, Taiwan; 6 Department of Safety Health and Environmental Engineering, Chung Hwa University of Medical Technology, Tainan, Taiwan; Azienda Ospedaliero Universitaria Careggi, ITALY

## Abstract

We investigated failure predictors for the planned extubation of overweight (body mass index [BMI] = 25.0–29.9) and obese (BMI ≥ 30) patients. All patients admitted to the adult intensive care unit (ICU) of a tertiary hospital in Taiwan were identified. They had all undergone endotracheal intubation for > 48 h and were candidates for extubation. During the study, 595 patients (overweight = 458 [77%]); obese = 137 [23%]) with planned extubation after weaning were included in the analysis; extubation failed in 34 patients (5.7%). Their mean BMI was 28.5 ± 3.8. Only BMI and age were significantly different between overweight and obese patients. The mortality rate for ICU patients was 0.8%, and 2.9% for inpatients during days 1–28; the overall in-hospital mortality rate was 8.4%. Failed Extubation group patients were significantly older, had more end-stage renal disease (ESRD), more cardiovascular system-related respiratory failure, higher maximal inspiratory pressure (MIP), lower maximal expiratory pressure (MEP), higher blood urea nitrogen, and higher ICU- and 28-day mortality rates than did the Successful Extubation group. Multivariate logistic regression showed that cardiovascular-related respiratory failure (odds ratio [OR]: 2.60; 95% [confidence interval] CI: 1.16–5.80), ESRD (OR: 14.00; 95% CI: 6.25–31.35), and MIP levels (OR: 0.94; 95% CI: 0.90–0.97) were associated with extubation failure. We conclude that the extubation failure risk in overweight and obese patients was associated with cardiovascular system-related respiratory failure, ESRD, and low MIP levels.

## Introduction

The prevalence of obesity is increasing worldwide and it has become a global public health threat. Obesity increases the risks of diabetes mellitus (DM), coronary artery disease (CAD), stroke, renal function impairment, respiratory dysfunction, and malignancy [1–4]. Because obesity has so many pathophysiologic effects upon all major organ systems and increases the prevalence of comorbidities, it is also significantly associated with higher risks of morbidity and mortality [5–7]. Therefore, an increasing number of obese patients need to be admitted to the intensive care unit (ICU) for critical care, and ICUs will inevitably have to treat a greater number of critically ill obese patients [8, 9].

Acute respiratory failure is one of the most frequent reasons that patients are transferred to the ICU. This condition is usually treated using endotracheal intubation with invasive mechanical ventilation, regardless of whether the patient is obese or normal weight [10–12]. A patient who has survived an acute condition that requires intubation must first be weaned from invasive ventilation. The critical care physician must then do a weaning test and decide the appropriate timing for extubation. However, even after a comprehensive evaluation, extubation fails for a significant percentage of patients, who then require reintubation. Several studies [13–17] have identified useful factors to accurately predict successful extubation. Several physiological parameters of pulmonary mechanisms [13,16,17], such as respiratory frequency-to-tidal volume (the rapid shallow breathing index [RSBI]), thoracic compliance, oxygenation, maximum occlusion pressure, and dynamic changes through the course of a spontaneous breathing trial (SBT), and airway protection capabilities, including mental status, cough strength, and secretion loads have been identified as possible predictors of successful extubation. However, the lung function of overweight patients is different from that of normal weight and underweight patients [18–21]. Thus, we aimed to determine what would predict the successful extubation of overweight and obese patients.

## Methods

### Patients and hospital setting

This study was done at the Chi Mei Medical Center, a 1288-bed tertiary medical center with 96 ICU beds for adults. The ICU is staffed by critical care attending physicians, senior residents, nurses, respiratory therapists, dietitians, physical therapists, and clinical pharmacists. Each team makes rounds at least once daily, and respiratory therapists manage all mechanical ventilation (MV) patients, including SBTs: T-piece or pressure support ventilation (PSV) for 30–120 minutes and weaning them from MV processes. All patients who required an invasive MV using an endotracheal tube for 48 hours and who were prepared for a scheduled extubation according to a weaning protocol and physicians’ judgment between January 2010 and December 2011 were eligible for inclusion. Each patient’s ICU admission weight and height were used to calculate their body mass index (BMI = weight in kg/height in m^2^). We measured weight in ICU patients with electronic weighing beds (Chang Gung Medical Technology Co., Ltd). Only adult (> 18 years old) patients with a BMI ≥ 25 kg/m^2^ were included in this study. For patients who required repeated intubation, only the first episode was included in the analysis.

### Variables measured

The medical records of all included patients were retrospectively reviewed and information was collected about age, gender, type of ICU, level of consciousness, intubation pathway (nose or mouth), why intubation was required, underlying diseases, and comorbidities, laboratory examination results, organ failure, intubation details, disease severity, length of ICU and hospital stays, reintubation rate, and mortality rate. After a patient had completed a successful SBT, the weaning parameters—respiratory rate, tidal volume, minute ventilation, RSBI, maximal inspiratory pressure (MIP), maximal expiratory pressure (MEP), and cuff leak test (CLT)—were measured when the patients were presumed ready for extubation. To accurately measure MIP, we measured 3 times with an interval of 5–6 seconds (2–3 breaths), and the value was recorded as the maximal data within these 3 checks. Additionally, we held the breath of unconscious and of uncooperative patients for 20 seconds from the end of inspiration to obtain the level of MIP. Thus, we improved the accuracy of MIP measurements. All data were routinely retrospectively collected and then analyzed. The Chi Mei Medical Center Institutional Review Board approved the study and specifically waived informed consent (IRB NO: 10206–005).

### Definitions

CLT scores were 2+ (audible flow without a stethoscope), 1+ (audible flow with a stethoscope), and N (negative; no audible flow with a stethoscope). Extubation failure was defined as reintubation within 48 hours of extubation. Overweight was defined as a BMI of 25.0–29.9 kg/m^2^ and obesity as a BMI of ≥ 30 kg/m^2^. Causes of respiratory failure were classified as the pulmonary system (upper airway obstruction, acute respiratory distress syndrome, chronic obstructive pulmonary diseases, pneumonia, malignant effusion, lobar collapse, asthma attack), cardiovascular system (congestive heart failure, pericarditis, cardiomyopathy, acute myocardial infarction, endocarditis), neurological system (status epilepticus, stroke), renal system (acute renal failure), gastrointestinal system, and others, as previously described [22,23].

### Statistical analysis

Continuous variables are reported as mean ± standard deviation (SD). Categorical variables are presented as *n* (%). In addition, the differences in baseline characteristics and clinical variables between the Successful and Failed Extubation groups were evaluated using a Student’s *t* test for continuous variables and a Pearson χ^2^ test for categorical variables. Those significantly associated with failed extubation in a univariate analysis (*P* < 0.05) were tested for interaction using a multivariate logistic regression analysis. Univariate and multivariate logistic regression analyses were used to calculate the odds ratios (ORs) and 95% confidence intervals (CIs) of significant variables to determine the association between predictive variables and successful extubation. SAS 9.4 for Windows (SAS Institute, Cary, NC, USA) was used for all analyses. Significance was set at *P* < 0.05 (two-tailed).

## Results

### Demographic characteristics

We enrolled 1934 patients who had been scheduled for a first-time extubation after more than 48 h of invasive MV (an endotracheal tube) after we had excluded eight patients without available BMI data (S1 File). More than half (55.9%; *n* = 1082) of the patients had a normal body weight (BMI = 18.5 to < 25), 257 (13.3%) were underweight (BMI < 18.5), and 595 (30.8%) were overweight or obese (BMI = ≥ 25). All patients were followed up until the primary outcome: in-hospital death or survival to discharge.

We analyzed 595 patients (mean age: 63.6 ± 15.8 years; range: 20–97 years; ≥ 65 years old: *n* = 283 [47.4%]; men: *n* = 403 [67.7%]; mean BMI: 28.5 ± 3.8) (Table 1) treated with > 48 h of MV and who underwent a planned extubation after weaning; the extubation of 34 patients (5.7%) failed. The most frequent causes of extubation failure were hemodynamic instability (*n* = 10), excess secretion (*n* = 8), upper airway obstruction (*n* = 8), oxygen failure (*n* = 6), and encephalopathy (*n* = 2). There were 458 (77%) overweight and 137 (23%) obese patients, 27 of whom had a BMI > 35. Pulmonary, cardiovascular, and neurological failures were the three most common etiologies of respiratory failure. The Acute Physiology and Chronic Health Evaluation (APACHE) II, Therapeutic Intervention Scoring System (TISS), and Glasgow coma scale scores upon ICU admission were 18.1 ± 6.8, 29.0 ± 7.9, and 11.2 ± 3.6, respectively. Endotracheal intubation via the oral route was done in > 95% of the patients. The most common underlying comorbidities were diabetes mellitus (DM) (*n* = 227 [38.2%]) and stroke (*n* = 174 [29.2%]). ICU stays were 10.4 ± 6.9 days long and hospital stays were 27.7 ± 22.4 days long. The overall in-hospital mortality rate was 8.4% (*n* = 50), the overall ICU rate was 0.8%, and the 28-day rate was 2.9%. BMI was significantly (*P* < 0.001) different between the overweight and obese patients, and obese patients were significantly younger than were overweight patients (60.5 ± 16.3 vs. 64.6 ± 15.6 years, *P* = 0.008) (Table 1).

**Table 1:** Characteristics of overweight (BMI: 25–29.9) and obese (BMI ≥ 30) patients treated with mechanical ventilation (MV) for > 48 h and undergoing planned extubation (*n* = 595).

Characteristics	Variable	Overweight	Obese	P*
		(BMI: 25–29.9)	(BMI ≥ 30)	
Male	403 (67.7)	315(68.8)	88(64.2)	0.319
Age (years)	63.6 ± 15.8	64.6 ± 15.6	60.5 ± 16.3	0.008
Elderly (≥ 65 years old)	283 (47.4)	220 (48.0)	62 (45.3)	0.568
Body mass index (BMI)	28.5 ± 3.8	27.0± 1.4	33.4 ± 5.0	< 0.001
Overweight (BMI: 25–29.9) [*n* (%)]	458 (77)			
Obese (BMI ≥ 30) [*n* (%)]	137 (23)			
Cause of respiratory failure				
Pulmonary system	144 (24.2)	105 (22.9)	39 (28.5)	0.184
Neurological system	141 (23.7)	105 (22.9)	36 (26.3)	0.418
Cardiovascular system	133 (22.4)	108 (23.6)	25 (18.2)	0.189
Gastrointestinal system	89 (15.0)	74 (16.2)	15 (10.9)	0.134
Renal system	39 (6.6)	26 (5.7)	13 (9.5)	0.114
Others	48 (8.1)	39 (8.5)	8 (6.6)	0.463
APACHE II	18.1 ± 6.8	18.2 ± 6.8	18.1 ± 6.9	0.869
Therapeutic Intervention Scoring System (TISS) Scale	29.0 ± 7.9	29.1 ± 8.0	28.8 ± 7.6	0.695
Glasgow coma scale	11.2 ± 3.6	11.3 ± 3.7	11.1 ± 3.5	0.703
Number of comorbidities	1.2 ± 0.9	1.6 ± 1.8	2.0 ± 2.9	0.994
Comorbidity				
Diabetes mellitus (DM)	227 (38.2)	166 (36.2)	61 (44.5)	0.080
Stroke	174 (29.2)	135 (29.5)	39 (28.5)	0.820
Coronary artery disease (CAD)	152 (25.5)	124 (27.1)	28 (20.4)	0.118
Cancer	82 (13.8)	63 (13.8)	19 (13.9)	0.973
Chronic obstructive pulmonary disease (COPD)	60 (10.1)	47 (10.3)	13 (9.5)	0.792
End-stage renal disease	51 (8.6)	37 (8.1)	14 (10.2)	0.432
Liver cirrhosis	18 (3.0)	14 (3.1)	4 (2.9)	0.935
Nasal endotracheal tube	26 (4.4)	21 (4.6)	5 (3.6)	0.638
Endotracheal tube size [median inner diameter: cm (IQR)]	7.5 (7.0–7.5)	7.5(7.0–7.5)	7.5(7.5–7.5)	
Intubation depth (cm)	22.6 ± 1.8	22.6 ± 1.9	22.6 ± 1.7	0.946
Intubation period before extubation (h)	180.9 ± 133.7	176.3 ± 133.9	196.4 ± 132.4	0.123
Length of intensive care unit (ICU) stay (days)	10.4 ± 6.9	10.3 ± 6.8	10.8 ± 7.1	0.414
Length of hospital stay (days)	27.7 ± 22.4	27.9 ± 235	27.1 ± 18.1	0.706
Reintubation within 48 h	34 (5.7)	28 (6.1)	6 (4.4)	0.443
In-hospital mortality rate	50 (8.4)	38 (8.3)	12 (8.8)	0.864

*Comparison between Overweight and Obese groups

Categorical variables are expressed as number *n* (%) and continuous variables as means ± standard deviation (SD) or median (interquartile range [IQR]).

APACHE: Acute Physiology and Chronic Health Evaluation.

### Comparison of patients in the Failed and Successful Extubation groups

There were many significant differences between the Failed and the Successful Extubation group patients. Those in the Failed Extubation group were older, had more end-stage renal disease (ESRD), and had a higher blood urea nitrogen (BUN) level (Table 2). They also had more respiratory failure because of cardiovascular system failure, had a less-negative MIP, and a less-positive MEP (Table 2).

**Table 2:** Comparison of Failed and Successful Extubation groups.

	Failed Extubation	Successful Extubation	*P*
	*n* = 34	*n* = 561	
Age (years)	69.5 ± 11.4	63.2 ± 16.0	0.004
Male [*n* (%)]	20 (58.8)	383 (68.3%)	0.253
Body mass index (BMI) (kg/m^2^)	28.0 ± 2.3	28.5 ± 3.9	0.422
Overweight (BMI: 25–29.9) [*n* (%)]	28 (82.4)	430 (76.6)	0.443
Obese (BM I≥ 30) [*n* (%)]	6 (17.6)	131 (23.4)	0.443
Medical department [*n* (%)]	14 (41.2)	255 (45.5)	0.626
Severity			
APACHE II	18.2 ± 7.3	18.1 ± 6.8	0.935
TISS Scale	30.7 ± 9.0	28.9 ± 7.8	0.191
Glasgow coma scale	11.7 ± 4.0	11.2 ± 3.6	0.409
Number of comorbidities	1.4 ± 1.0	1.2 ± 0.9	0.343
Comorbidity [*n* (%)]			
End-stage renal disease	17 (50.0)	34 (6.1)	< 0.001
Diabetes mellitus (DM)	15 (44.1)	212 (37.8)	0.461
Stroke	12 (35.3)	140 (25.0)	0.180
Coronary artery disease (CAD)	9 (26.5)	165 (29.4)	0.714
Cancer	5 (14.7)	77 (13.7)	0.872
COPD	3 (8.8)	57 (10.2)	0.802
Liver cirrhosis	0 (0)	18 (3.2)	0.289
Cause of respiratory failure [*n* (%)]			
Pulmonary system	6 (17.6)	138 (24.6)	0.358
Cardiovascular system	15 (44.1)	118 (21.0)	0.002
Neurological system	8 (23.5)	133 (23.7)	0.981
Renal system	3 (8.8)	36 (6.4)	0.582
Gastrointestinal system	2 (5.9)	87 (15.5)	0.126
Others	0 (0)	48(8.6)	0.075
Intubation period before extubation (h)	188.6 ± 112.6	180.4 ± 134.9	0.729
Parameter before extubation			
Respiratory rate (breaths/min)	18.7 ± 5.4	17.9 ± 5.1	0.401
Tidal volume (mL)	429.9 ± 162.3	459.2 ± 161.7	0.312
Minute ventilation (L/min)	9.30 ± 4.08	9.38 ± 3.11	0.889
RSBI (breaths/min/L)	57.6 ± 27.0	53.6 ± 26.4	0.402
MIP (cm H_2_O)	-30.7 ± 12.0	-41.7 ± 15.0	0.001
MEP (cm H_2_O)	32.8 ± 9.1	65.8 ± 30.4	0.001
Cuff leak test			
N	2 (5.9)	18 (3.2)	0.401
+	6 (17.6)	81 (14.4)	0.607
++	26 (76.5)	432 (77.0)	0.943
Laboratory examinations			
pH	7.441 ± 0.048	7.446 ± 0.046	0.534
PaCO_2_ (mmHg)	37.1 ± 5.2	39.0 ± 6.2	0.082
PaO_2_ (mmHg)	89.3 ± 27.6	91.7 ± 23.6	0.577
PaO_2_/FiO_2_ (mmHg)	296.3 ± 112.2	326.7 ± 90.9	0.070
Hemoglobin (Hb) (g/dl)	11.1 ± 2.0	11.1 ± 2.0	0.960
Blood urea nitrogen (BUN) (mg/dl)	43.5 ± 39.3	28.5 ± 23.9	0.040
Creatinine (mg/dl)	2.40 ± 2.17	1.62 ± 2.10	0.058
Albumin (mg/dl)	2.9 ± 0.5	2.8 ± 0.6	0.264

Categorical variables are expressed as *n* (%) and continuous variables as means ± standard deviation (SD).

APACHE: Acute Physiology and Chronic Health Evaluation; TISS: Therapeutic Intervention Scoring System; COPD: chronic obstructive pulmonary disease; RSBI: rapid shallow breathing index; MIP: maximum inspiratory pressure; MEP: maximum expiratory pressure; PaCO_2_: partial pressure of carbon dioxide in arterial blood; PaO_2_: partial oxygen tension in arterial blood; FiO_2_: fractional concentration of oxygen in inspired gas.

Failed Extubation group patients had higher mortality rates than did Successful Extubation group patients (Table 3). They were also transferred to the Respiratory Care Center and Respiratory Care ward because it was difficult to wean them from MV Consequently, they had longer ICU and hospital stays, and higher hospital costs than did patients in the Successful Extubation group (Table 3).

**Table 3:** Outcomes of Failed and Successful Extubation groups.

Variable	All	Failed Extubation	Successful Extubation	*P*
	*n* = 595	*n* = 34	*n* = 561	
Intensive care unit (ICU) mortality	5 (0.8)	3 (8.8)	2 (0.4)	< 0.001
28-day mortality	17 (2.9)	5 (14.7)	12 (2.1)	< 0.001
Transferred to respiratory care center	45 (7.6)	25 (73.5)	20 (3.6)	< 0.001
Transferred to respiratory care ward	4 (0.7)	3(8.8)	1 (0.2)	< 0.001
ICU stay (days)	10.4 ± 6.9	14.4 ± 7.2	10.2 ± 6.8	< 0.001
Hospital stay (days)	27.7 ± 22.4	36.5 ± 24.1	27.2 ± 22.2	0.018
Hospital cost (NT$10,000)	35.7 ± 31.6	49.8 ± 31.5	34.9 ± 31.4	0.007

Categorical variables are expressed as *n* (%) and continuous variables as means ± standard deviation (SD).

NT$: New Taiwan dollars (US$1 = ca. NT$32.5).

### Multivariate analysis

Multivariate logistic regression showed that cardiovascular system failure-induced respiratory failure (OR: 2.60; 95% CI: 1.16–5.80), ESRD (OR: 14.00; 95% CI: 6.25–31.35), and lower MIP levels (OR: 0.94; 95% CI: 0.90–0.97) were associated with failed extubation (Table 4).

**Table 4:** The predictors of failed planned-extubation within 48 h.

Variable	Univariate Analysis			Multivariate Analysis		
	Odds Ratio	95% CI	*P*	Odds Ratio	95% CI	*P*
Age	1.03	1.00–1.05	0.025			
Cardiovascular failure	2.96	1.46–6.01	0.003	2.60	1.16–5.80	0.020
End-stage renal disease	15.50	7.28–33.03	< 0.001	14.0	6.21–31.35	< 0.001
Maximal inspiratory pressure	0.93	0.90–0.97	0.001	0.94	0.90–0.97	0.001
Maximal expiratory pressure	0.980	0.96–0.99	0.006			

## Discussion

Our most important finding was that the MIP level was significantly associated with the outcome of extubation. Although impaired respiratory muscle loading might be induced by dysfunctional respiratory system mechanics and high ventilation demands, our finding indicated that extubation failure might be closely related to the power of respiratory muscle to overcome the burden of a large body size. In contrast, the RSBI, which is most commonly used to evaluate the ability of patients to breathe spontaneously and to be successfully weaned from MV, was not significantly different between the Failed and Successful Extubation groups. Studies [24, 25] on neurology patients report that the RSBI does not predict extubation failure. Although others [26–28] have identified many variables, e.g., age, mental status, oxygenation impairment, and the severity of acute illnesses, as risk factors for extubation failure and reintubation, we did not find that these variables were independent predictors of reintubation. This might be partly because of our unique study population: overweight and obese patients. It indicates that more studies are needed to investigate and establish prediction models of failed and successful extubation in specific populations.

We also found that ESRD was independently associated with extubation failure. A recent study [29] on surgical ICUs reported that an elevated BUN level (> 8.2 mmol/L) was an independent risk factor for reintubation (OR: 3.66; 95% CI: 1.97–6.80) in patients with a median BMI of 26.9 (no reintubation group; *n* = 699) and with a median BMI of 26.2 (reintubation group; *n* = 65). Another study [30] reported that acute kidney injury (OR: 2.98; 95% CI: 2.13–4.02) was associated with reintubation after coronary artery bypass grafting surgery. Overall, renal failure can be a risk factor of extubation failure in overweight patients and other populations.

A third important finding is that the risk of extubation failure was higher when the cause of respiratory failure was a cardiovascular system failure. In the present study, more than 40% of our patients with extubation failure had cardiovascular system failure that had required the patients to be intubated. Obesity itself is an important risk factor for the development of cardiovascular diseases [31]; therefore, physicians should more carefully assess the timing of extubation, especially for obese patients with cardiac failure. However, additional studies are required to confirm this finding.

Finally, our patients with a failed extubation, like those in other studies [22,26,32], had worse outcomes, including higher mortality and longer hospital stays. However, the extubation failure rate in the present study was only 5.7%, significantly lower than those in two of those studies [26,32]. The difference might be attributable to different study designs and weaning protocols. Our finding suggests, however, that it is possible to select patients who can be successfully extubated after a comprehensive assessment, including a conventional weaning profile, underlying disease, and the cause of acute respiratory failure. It is also possible that the low failure rate in our study was caused by delayed extubation (the average duration of MV was 7–8 days in the present study). However, delayed extubation is also reported to be associated with worse outcomes, such as pneumonia, length of stay in the ICU and in the hospital, and mortality [33]. Therefore, it is important for physicians to weigh risks versus benefits between delayed and early extubation. We recommend that for overweight and obese patients, physicians should carefully evaluate MIP level, the cause of respiratory failure, and comorbid ESRD before scheduling an extubation.

This study has some limitations. It was done in a single tertiary medical center, the final decision to extubate was made by ICU physicians. Extubation can be delayed until patients show improvement in all variables. Therefore, our findings might not be generalizable to other populations. Because this is the first report that focuses on the risk factors of extubation failure in overweight and obese patients, it offers some useful insights. Although we tried to adjust for all possible confounding factors in this retrospective study, we might have missed some other cofactors, such as previously prescribed and taken sedatives. Further investigations are warranted.

## Conclusion

The risk of extubation failure in overweight and obese patients was associated with MIP level, cardiovascular system failure-related intubation, and underlying ESRD.

## Supporting information

S1 File Dataset of study subjects. (XLS)
